# A diet including xanthan gum triggers a pro-inflammatory response in Wistar rats inoculated with Walker 256 cells

**DOI:** 10.1371/journal.pone.0218567

**Published:** 2019-06-18

**Authors:** Alessandra B. Silva Rischiteli, Nelson I. P. Neto, Karina Gascho, Marcela Carnier, Danielle A. de Miranda, Fernanda P. Silva, Valter T. Boldarine, Marília Seelaender, Eliane B. Ribeiro, Lila M. Oyama, Claudia M. Oller do Nascimento

**Affiliations:** 1 Universidade Federal de São Paulo, Escola Paulista de Medicina, Departamento de Fisiologia, São Paulo, Brasil; 2 Department of Cell and Developmental Biology, Institute of Biomedical Sciences, University of São Paulo, São Paulo (SP), Brasil; Temple University School of Medicine, UNITED STATES

## Abstract

**Objective:**

The aim of this study was to evaluate the effect of adding xanthan gum to the diet of rats on the production of cytokines and pro-inflammatory factors and on tumor development in rats inoculated with Walker 256 tumor cells.

**Methods:**

Fifty-six rats were divided into 4 groups: control diet (C), control diet with tumor (TC), xanthan gum diet (XG), xanthan gum diet with tumor (TXG).

**Results:**

The ingestion of xanthan gum promotes changes in cytokine content: increasing IL-6 TNF-α and IL-10 in retroperitoneal adipose tissue compared to the control group; and increasing TNF-α in the mesenteric adipose tissue compared to the C and TXG groups. On the contrary, the addition of xanthan gum to the diet did not affect the development of Walker 256 tumors in rats.

**Conclusion:**

The continuous use of xanthan gum triggered a pro-inflammatory response, promoting an increase in pro-inflammatory cytokines in the adipose tissue, but it did not have an effect on the tumor development in the animals inoculated with Walker 256 tumor cells.

## Introduction

People with dysphagia ingest xanthan gum daily and continuously because it is widely used as a thickening agent to adjust food consistency and maintain safe swallowing, without the risk of bronchoaspiration [1] [2]. Two articles published in 2012 by Woods et al. [3] and Beal et al. [4], describe that the continuous use of xanthan gum in neonates caused the development of necrotizing enterocolitis, a highly inflammatory process.

Chronic pro-inflammatory status has been associated with an increased risk of cancer, since a wide variety of chronic inflammatory conditions predispose normal cells to neoplastic transformation. In general, the longer the inflammation persists, the higher is the risk of developing cancer [5].

Currently, cancer is one of the main causes of death. Its etiology is multifactorial and may be associated with a combination of genetic and environmental factors related to lifestyle. Systemic inflammation is recognized as a hallmark in the development and progression of cancer [6] [7].

Inflammation is a process that includes injury, repair and resolution. In response to tissue damage, a multifactor network of chemical signals initiates and maintains a response to repair the affected tissue. This involves the activation and targeted migration of leukocytes (neutrophils, monocytes and eosinophils) from the venous system to the sites of damage, in addition to mast cells and neutrophils, which also play an important role in repair of these lesions. Cell proliferation per se is known to not be a determining factor in tumor development. However, sustained cell proliferation in an rich environment rich in inflammatory cells, growth factors, and DNA-damaging agents certainly potentiates or promotes the increased risk of neoplasms [8].

Pro-cancer events activate two major cell signaling pathways: nuclear kappa factor (NFkB) and signal transducer and activator of transcription 3 (STAT3). Both transcription factors are linked to initiating inflammation and cell growth factors, angiogenesis, and cytokine / chemokine regulation [9].

The NFkB and STAT3 pathways are central pathways in inflammation and tumorigenesis. Both are activated by a wide variety of events associated with tumors such as growth factors (EGF), factor alpha 1 (HIF1) induced hypoxia, an acidic microenvironment, hyperglycemia (diabetes and insulin resistance) and cytokines alterations. TNF-α is one of the most powerful activators of NFκB, which explains the strong association between high levels of TNF-α and the aggressive behavior of several types of TNF-α tumors [10] [11].

Adipokines have also been reported to be involved in tumor growth. In addition, adipose tissues in a state of hypoxia, wich occurs in obesity, recruit and alter the phenotype of macrophages; these macrophages may promote tumor progression by the secretion of inflammatory cytokines, such as TNF-α and IL-6 [12].

In 2009, Takeushi et al. [13], treated TLR4^+ / +^ and TLR4^- /—^mice with 1mg of xanthan gum every 5 days, until one day prior to the inoculation of B16Kb and MBT-2 melanomic cells. They found that treatment with xanthan gum only reduced tumor growth in TLR4^+ / +^ mice. The authors also detected an increased production of TNF-α in the peritoneal macrophages of animals inoculated with xanthan gum. They concluded that xanthan gum has an antineoplastic effect by stimulating macrophages to secrete TNF-α through the activation of TLR-4.

As reported by Sumantran and Tillu [14], acute inflammatory processes have anti-infection and anticancer effects. However, as previously described, chronic inflammation potentiates the risk and development of tumor cells.

In our opinion, the protocol used by Takeushi et al [13] induced momentary pro-inflammatory states, alternating with an absence of stimulation due to the 5 day xanthan gum administration schedule and not a chronic pro-inflammatory state, which in the literature is clearly associated with the risk of tumor development.

Thus, the primary objective of this study was to evaluate the effect of xanthan gum in the diet of rats on the production of cytokine and pro-inflammatory factors, as well as on tumor development in rats inoculated with Walker 256 tumor cells.

## Material and methods

### Animals and treatments

Fifty-six 30-day old male Wistar rats. Animal experiments were performed in accordance with the Brazilian Guideline for Care and Use of Animals for Scientific Purposes and Teaching, which are prescribed by the National Council of Animal Experimentation and approved by the Experimental Research Committee of the Universidade Federal de São Paulo (CEUA n° 6264030915). The experimental protocol was performed twice to confirm the reproducibility of our model.

During the experimental period, the animals were maintained collectively in a polypropylene cage under controlled temperature (23 ± 1°C) and lighting conditions (lights on from 6 a.m. to 6 p.m.). After 7 days of adaptation, the rats were split into two groups. Over a period of 8 weeks, the control group received a control diet + water ad libitum (C) (n = 28), whereas the treatment group received the control diet added with xanthan gum (185mg/100g) + water (XG) (n = 28). The control diet was Nuvilab (Nuvilab, Brazil, 2.79 kcal.g^-1^).

After 8 weeks, the rats were randomly divided into four groups: control diet non-inoculated (C), diet added with xanthan gum non-inoculated (XG), control diet inoculated with Walker 256 tumor cells (TC), and diet added with xanthan gum inoculated with Walker 256 tumor cells (TXG). The rats assigned to the Walker 256 tumor cell groups were subcutaneously injected with Walker 256 tumor cells (2 × 10^7^ cells) in the right flank.

The Walker 256 tumor cell line was chosen since it is a solid adenocarcinoma models frequently used in rodent model studies.

Body weight and food consumption were measured on a weekly basis and the total food intake was calculated over the 10 weeks of treatment.

### Oral glucose tolerance test (OGTT)

Seven days post Walker 256 tumor cell inoculation the four groups were subjected to an OGTT. After 8 h of fasting, blood was collected from the rat’s tail veins analyze basal glucose concentration. A glucose solution (2 g/kg) was then administrated by gavage and blood samples were collected after 15, 30, 45, 60, 90, and 120 min to measure glucose concentration using a glucose analyzer (AccuCheck Roche). During the entire OGTT period the animals were kept without anesthesia and unconfined.

### Experimental procedures

Fourteen days post tumor inoculation, the animals fasted for 8 h before being euthanatized under anesthesia with ketamine (70 mg/kg) and xylazine (10 mg/kg). This period was selected based on the mean survival time of animals, after Walker 256 cells inoculation, reported by Fenner et al., 2015 [15]. Trunk blood was collected and centrifuged to separate the serum, which was stored at −80°C. The epididymal (EPI), mesenteric (MES), and retroperitoneal (RET) white adipose tissues, liver, gastrocnemius muscle (GAST), and tumors were removed, weighed, and immediately frozen in liquid nitrogen before being stored at −80°C. The carcasses were eviscerated and stored at −20°C.

### Carcass lipid and protein content

Carcass lipid and protein content was determined as described previously by Carnier et al., 2018 [16]. The results are expressed in grams of lipid or protein per 100 g of carcass.

## Biochemical and hormonal serum analyses

Glucose, triacylglycerols, total cholesterol, and HDL-cholesterol serum concentrations were measured using a commercially available enzymatic colorimetric kit (Labtest, Brazil). Insulin, adiponectin and leptin concentrations were determined using specific enzyme-linked immunosorbent assay kits (Millipore and R&D Systems, respectively).

## Total protein extraction

After euthanasia, the previously mentioned tissues were rapidly removed and frozen. The tissues were homogenized in 800 μL of chilled extraction buffer (100 mM Trizma Base, pH 7.5; 10 mM EDTA; 100 mM NaF; 10 mM Na_4_P_2_O_7_; 10 mM Na_3_VO_4_; 2 mM PMSF; and 0.1 mg/mL aprotinin). Following homogenization, 80 μL of 10% Triton X-100 was added to each sample. The samples were kept on ice for 30 min and then centrifuged (20817 *g*, 40 min, 4°C) and the supernatant was used for ELISA. Total tissue protein concentration was determined with used the Bradford assay (Bio-Rad, Hercules, California), and bovine serum albumin was used as a reference protein.

### IL-6, IL-10, IL-1β, and TNF-α protein levels determined by ELISA

IL-6, IL-10, IL-1β, and TNF-α content in the tissues was analyzed by ELISA (DuoSet ELISA, R&D Systems, Minneapolis, MN, USA), following the manufacturer’s instructions. All samples were run in duplicate.

### Statistical analysis

The distribution of data was verified using Levene’s test of equality of error variances and Shapiro–Wilk tests. Parametric variables are expressed as means and standard error, whereas non-parametric variables are expressed as median [minimum–maximum]. The statistical significance of the difference between the means of parametric variables was assessed using Unpaired Student *t* test or two-way analysis of variance, followed by the *post-hoc* Bonferroni test. The Kruskal–Wallis test was used for non-parametric variables, followed by Mann–Whitney–Wilcoxon test. Differences were considered to be statistically significant when p < 0.05.

## Results

### Body weight changes, total food intake, tissue weight, and carcass lipid and protein contents

Body weight changes, total food intake, tissue weights, and the lipid and protein contents of carcasses of all groups were similar (Tables 1 and 2).

**Table 1:** Body weight changes (g) and total body weight gain (g), total food intake (g) and food efficiency of rats fed the control diet non-inoculated (C), diet added with xanthan gum non-inoculated (XG), control diet inoculated with Walker 256 tumor cells (TC) and diet added with xanthan gum inoculated with Walker 256 tumor cells (TXG) during 10 weeks of diet treatment.

	C (22)		XG (24)	
Initial ^2^	181.15 ± 3.84		177.56 ± 3.74	
Week 1^2^	232.02 ± 4.12		227.27 ± 3.72	
Week 2^2^	283.09 ± 3.55		280.78 ± 3.86	
Week 3^2^	336.95 ± 4.21		330.21 ± 4.32	
Week 4^2^	377.49 ± 5.42		372.87 ± 4.63	
Week 5^2^	416.29 ± 6.35		414.50 ± 5.57	
Week 6^2^	439.61 ± 6.90		437.42 ± 6.10	
Week 7^2^	465.49 ± 8.01		460.43 ± 6.63	
Week 8^2^	483.81 ± 8.42		480.47 ± 6.92	
AFTER TUMOUR WALKER 256 INOCULATION				
	C (11)	CT (11)	XG (12)	TXG (12)
2º. Day^1^	484.06 ± 11.90	496.28 ± 12.19	483.46 ± 10.00	489.61 ± 11.09
4°. Day^1^	490.68 ± 12.90	504.70 ± 12.76	490.24 ± 10.32	497.49 ± 11.24
6°. Day^1^	493.34 ± 14.53	509.16 ± 13.95	494.72 ± 11.35	500.68 ± 11.47
8°. Day^1^	488.77 ± 12.68	504.51 ± 12.98	489.98 ± 10.59	499.01 ± 11.00
10°. Day^1^	492.50 ± 12.65	513.34 ± 14.18	497.35 ± 11.64	503.17 ±11.47
12°. Day^1^	498.80 ± 12.83	513.33 ± 15.66	504.16 ± 11.54	509.48 ± 11.20
140°. Day^1^	490.54 ± 13.04	517.00 ± 14.41	494.38 ± 12.10	504.19 ± 11.44
Body Weight without tumor^2^		490.19 ± 17.44		473.10 ± 13.83
Total body weight gain (g)^1^	306.96 ± 16.08	329.51 ± 19.78	312.97 ± 11.91	330.48 ± 13.01
Total food intake (g)^1^	1792.76 ± 23.72	1862.13 ± 36.23	1935.78 ± 19.07	1878.21 ± 21.49
Food efficiency^1^	5.96 ± 0.24	5.82 ± 0.30	6.29 ± 0.27	5.79 ± 0.24

Note: The number in parentheses refers to the sample value. Data are expressed as means ± SEM.

^1^ Two way ANOVA followed Bonferroni.

^2^ Unpaired Student t test.

**Table 2:** Tissues weight (g), lipid and protein contents of carcasses [g/100 g body weight] of rats fed the control diet non-inoculated (C), diet added with xanthan gum non-inoculated (XG), control diet inoculated with Walker 256 tumor cells (TC) and diet added with xanthan gum inoculated with Walker 256 tumor cells (TXG).

	C (11)	TC (11)	XG (12)	TXG (12)
Epididymal adipose tissue^1^	8.75 ± 0.60	9.12 ± 1.04	7.49 ± 0.86	7.64 ± 0.82
Retroperitoneal adipose tissue^1^	8.40 ± 0.88	8.14 ± 1.01	8.85 ± 1.15	7.48 ± 0.88
Mesenteric adipose tissue^1^	5.76 ± 0.63	5.82 ± 0.68	5.96 ± 0.73	4.66 ± 0.53
Adiposity^1^	22.91 ± 1.82	23.07 ± 2.40	22.30 ± 2.36	19.89 ± 2.06
Gastrocnemius muscle^1^	2.23 ± 0.05	2.19 ± 0.08	2.26 ± 0.06	2.06 ± 0.08
Liver^1^	14.02 ± 0.44	14.43 ± 0.35	14.22 ± 0.40	15.07 ± 0.38
Tumor^2^		22.32 ± 3.78		31.09 ± 4.99
Carcass lipid content (g/100g b.w.)^1^	12.06 ± 2.34	9.75 ± 1.27	7.70 ± 1.62	8.61 ± 0.78
Carcass protein content (g/100g b.w.)^1^	31.28 ± 2.95	34.14 ± 4.64	30.44 ± 2.20	28.94 ± 4.14

Note: The number in parentheses refers to the sample value. Data are expressed as means ± SEM.

^1^ Two way ANOVA followed Bonferroni.

^2^ Unpaired Student t test.

### OGTT and serum parameters

The addition of xanthan gum for 7 days post tumor Walker 256 cell inoculation did not alter OGTT results. However, the serum glucose concentration 14 days after tumor inoculation was lower in the TC and TXG groups than the C and XG (Tables 3 and 4). The area under the curve (AUC) was similar in all the groups.

**Table 3:** Oral glucose tolerance test (OGTT: mg/dL) and area under the curve (AUC) of rats fed the control diet non-inoculated (C), diet added with xanthan gum non-inoculated (XG), control diet inoculated with Walker 256 tumor cells (TC) and diet added with xanthan gum inoculated with Walker 256 tumor cells (TXG) after 7 days of inoculation and 8 hours fast.

Time in minutes	C (5)	TC (6)	XG (6)	TXG (6)
0’	81.00 ± 2.55	81.00 ± 2.36	85.67 ± 4.49	75.40 ± 2.43
15’	137.40 ± 4.91	115.40 ± 3.62	115.33 ± 10.14	131.200 ± 5.94
30’	154.60 ± 5.49	131.60 ± 4.21	146.33 ± 6.11	136.20 ± 5.18
60’	133.80 ± 6.01	131.40 ± 4.21	128.67 ± 6.34	130.00 ± 2.14
90’	125.40 ± 4.13	124.40 ± 2.36	122.17 ± 4.44	115.80 ± 5.41
120’	111.80 ± 3.06	113.40 ± 3.77	106.33 ± 5.35	104.80 ± 4.46
AUC	15600 ± 395.26	14674.50 ± 271.50	14785 ± 685.68	14547 ± 255.19

Note: The number in parentheses refers to the sample value. Data are expressed as means ± SEM.

**Table 4:** Serum concentration of insulin, adiponectin, triacylglycerol, total cholesterol. HDL-cholesterol, glucose and leptin of rats fed the control diet non-inoculated (C), diet added with xanthan gum non-inoculated (XG), control diet inoculated with Walker 256 tumor cells (TC) and diet added with xanthan gum inoculated with Walker 256 tumor cells (TXG).

	C (11)	TC (11)	XG (12)	TXG (12)
Glucose(mg/dL)	143.54 *	120.57	147.04 *	116.12
	± 5.87	± 9.15	± 5.17	± 8.14
Triacylglycerol (mg/dL)	113.51	131.73	116.95	163.04 ^$^
	(86.96–141.57)	(96.49–308.26)	(91.30–173.62)	(91.74–247.75)
Total cholesterol (mg/dL)	87.06	109.95	87.93	115.71
	(64.83–135.60)	(67.93–140.84)	(69.31–130.37)	(66.21–142.93)
HDL-cholesterol(mg/dL)	38.00	36.04	43.99 ^#^	31.87
	± 1.98	± 3.68	± 2.88	± 4.87
Insulin (ng/mL)	1.20	0.92	1.00	0.33
	(0.44–2.44)	(0.40–1.56)	(0.24–2.07)	(0.032–1.80)
Leptin (ng/mL)	4.49	6.71	3.59	3.20
	(0.23–8.31)	(1.00–17.17)	(1.54–20.17)	(0.89–7.56)
Adiponectin (μg/mL)	3.79	2.94	3.77	2.13
	(2.33–4.51)	(0.14–16.67)	(1.69–7.75)	(0.67–6.50)

Note: The number in parentheses refers to the sample value. For parametric measure, values are expressed as means ± standard error of the mean; for non-parametric measure values are expressed as the median [minimum–maximum]. The statistical difference between the groups was symbolized with:

* Significant at p value <0.05 groups C and XG *versus* TC and TXG

^$^ Significant at p value <0.05 group TXG *versus* C and XG

^#^ Significant at p value <0.05 group XG *versus* TXG

Triacylglycerol was higher and HDL-cholesterol was lower in the TXG group than the XG group. Insulin, adiponectin and leptin serum concentrations did not differ between the groups (Table 4).

### Tissue cytokine content

The cytokine content in the tissues are presented in Table 5. The most significant result was the increase in IL-6, TNF-α and IL -10 in the retroperitoneal adipose tissue of the XG group compared to that of the control group and increase in TNF-α in the mesenteric adipose tissue of the XG group compared to that of the C and TXG groups.

**Table 5:** Tissues cytokines content (pg/mg of protein) of rats fed the control diet non-inoculated (C), diet added with xanthan gum non-inoculated (XG), control diet inoculated with Walker 256 tumor cell (TC) and diet added with xanthan gum inoculated with Walker 256 tumor cell (TXG).

	C (5–11)	TC (5–11)	XG (5–12)	TXG (5–12)
	LIVER			
IL-10	26.24	24.31	28.10	20.31
	(14.98–44.52)	(17.44–65.85)	(15.37–57.64)	(13.42–104.01)
IL-6	99.26	91.4	112.23	84.33
	(61.97–129.10)	(73.04–163.63)	(56.25–173.52)	(55.46–291.04)
IL1-β	50.91	56.49	44.02	63.63
	(32.78–93.96)	(28.90–93.60)	(18.57–139.17)	(32.22–156.22)
TNF-α	48.66	49.78	56.02	67.23
	(32.51–140.41)	(39.15–69.21)	(35.11–106.83)	(23.24–120.94)
	RETROPERITONEAL			
IL-10	99.36	183.81	170.80*	78.83
	(28.15–159.11)	(52.36–363.85)	(70.58–442.44)	(23.34–296.04)
IL-6*	737.78	1337.91	1697.78^$^	510.74
	(157.94–1037.81)	(339.34–2217.57)	(424.03–2616.39)	(177.73–1949.80)
IL1-β	142.65	144.87	136.30	88.73
	(71.66–214.78)	(30.79–484.96)	(64.92–665.99)	(71.30–194.80)
TNF-α	474.80	968.25	972.83^#^	306.60
	(94.99–884.04)	(190.51–1754.53)	(288.73–1751.47)	(97.30–1327.23)
	EPIDIDYMAL			
IL-10	27.94	28.47	44.07	39.60
	(9.27–52.17)	(4.30–73.69)	(6.96–192.25)	(14.94–92.82)
IL-6	115.24	143.84	179.71	183.46
	(29.28–246.99)	(7.35–319.68)	(12.20–945.27)	(13.01–465.92)
IL1-β	43.46	61.76	84.30	49.60
	(21.09–183.68)	(22.02–118.39)	(23.43–221.39)	(18.87–148.13)
TNF-α	111.84	99.48	175.34	169.06
	(40.83–154.94)	(17.47–276.11)	(25.61–726.59)	(43.13–426.77)
	MESENTERIC			
IL-10	17.14	15.47	18.11	16.04
	± 1.76	± 3.38	± 2.18	± 2.88
IL-6	97.74	51.79	155.12	54.22
	(43.26–120.46)	(28.46–188.66)	(123.12–197.75)	(17.76–71.21)
IL1-β	79.37	62.99	101.65	41.60
	(37.65–93.11)	(29.82–127.30)	(90.10–140.01)	(15.81–47.70)
TNF-α	305.73	190.45	496.45**	219.07
	(165.78–381.35)	(122.55–580.76)	(468.53–546.37)	(53.97–285.91)
	GASTROCNEMIUS MUSCLE			
IL-10	2.53	2.40	3.25	2.57
	(0.96–4.78)	(1.11–5.81)	(0.24–16.44)	(1.29–5.91)
IL-6	22.02	18.60	27.89	30.15^$ $^
	(10.97–40.64)	(13.74–24.62)	(9.32–70.04)	(15.82–41.49)
IL1-β	13.57^##^	14.45^##^	11.68	9.10
	± 1.62	± 1.83	± 1.50	± 1.24
TNF-α	11.90	8.69	16.09	11.21
	(4.98–20.99)	(4.16–21.79)	(3.31–65.34)	(5.49–18.84)
	TUMOR			
IL-10		2.18		1.59
		± 0.58		± 0.51
IL-6		14.20a		10.74
		(9.53–31.93)		(7.38–20.65)
IL1-β		54.09		51.14
		± 7.65		± 8.20
TNF-α		15.95		9.77
		± 5.32		± 1.16

Note: The number in parentheses refers to the sample value. For parametric measure, values are expressed as means ± standard error of the mean; for non-parametric measure values are expressed as the median [minimum–maximum]. The statistical difference between the groups was symbolized with:

* Significant at p value <0.05 group XG *versus* C

^$^ Significant at p value <0.05 group XG *versus* C

^#^ Significant at p value <0.05 group XG *versus* C

** Significant at p value <0.05 group XG versus C and TXG

^$ $^ Significant at p value <0.05 group TXG versus TC

^##^ Significant at p value <0.05 groups C and CT versus GX and TXG

L-6 in the gastrocnemius muscle was higher in the xanthan gum diet (TXG) group animals inoculated with Walker 256 tumor cells, compared to TC group animals inoculated with Walker 256 tumor cells. The IL-1β content of the gastrocnemius muscle was also lower in the XG and TXG groups than in the C and TC groups.

Intake of xanthan gum or inoculation with Walker tumor cells did not induce any changes in the analyzed cytokine content of the liver, tumor or epididymal adipose tissues.

## Discussion

This study presents new results on the effect of adding xanthan gum to the daily diet of rats on the inflammatory profile of tissues and the development of Walker 256 tumors.

The addition of xanthan gum to the diet altered the cytokine content in some of the tissues studied; promoting a higher proinflammatory effect compared to that in the C and XG groups. On the other hand, the addition of xanthan gum to the diet did not affect the development of Walker 256 tumors in the rats. However, when we compared the XG group to the TGX group, we found that tumor inoculation caused changes in plasma biochemical parameters and decreased inflammatory cytokines in the mesenteric adipose tissue.

There were no changes in body weight or total dietary intake due to the xanthan gum (C vs. XG) or Walker 256 cells (C vs. TC and XG vs. TXG) for duration of the experiment. Similar results were observed by das Neves et al. [17] when they inoculated Walker 256 tumor cells into the bone marrow of Wistar rats. On the other hand, Batista et al [18] found reductions in the body weight after the rats were inoculated with Walker 256 tumor cells using the same protocol as this study, but the weights of the animals were also different at the time of inoculation. In our study the animals weighed 2 times more than those used in Batista's research. A comparison of our results to the literature suggests that body weight changes after inoculation with Walker 256 tumor cells depends partly on of the site of inoculation and the weight of the animals before the procedure, but also on the duration of the study.

No significant difference in tissue weights or fat and protein contents of the carcasses was observed between the groups, consistent with, the similarity in their body mass gain. Demonstrating that neither xanthan gum nor inoculation with Walker 256 cells altered body composition or food intake within 14 days.

Glucose tolerance at baseline (time zero),15, 30, 60, 90 and 120 min did not differ significantly between the groups. However, when analyzing serum glucose concentration 14 days post tumor cell inoculation, the glycaemia of the TXG and TC animals was lower that of in the C and XG animals, showing that the tumors resulted in a reduction in blood glucose. In 1930 Warburg [19] found that the survival of tumor cells depends on their glucose supply, that is, they require a high level of glycolysis. In 1967 [20] glycolysis was studied in rats inoculated with Walker 256 tumor cells. Thus, the reduction of glycaemia in TXG and TC may have occurred due to the increased use of glucose by the tumor cells.

Insulinemia and total cholesterol, leptin and adiponectin serum concentrations of the groups were similar. On the contrary, the HDL-cholesterol was reduced and TAG concentration was elevated in the animals that developed Walker 256 tumors and had xanthan gum added to their diet.

TAG is a complex fatty acid. In order for fatty acids to be released into the bloodstream, lipoprotein lipase (LPL) must act on TAGs of TAG-rich lipoproteins or hormone-sensitive lipase must act on the TAGs of adipose tissue. LPL is an enzyme in the lipase family that hydrolyzes TAG molecules found in the lipoprotein particles. As fatty acids are liposoluble compounds, they rapidly diffuse into plasma hydrolysis sites by binding to acceptor molecules capable of solubilizing them [21]. When lipid metabolism was studied in rats with Walker 256 tumors by Seelaender et al. in 1996 [22], the researchers argued that altered lipid metabolism may be associated with decreased LPL activity and hence hypertriglyceridemia. In a more recent study [23] plasma lipoproteins were altered in patients with neoplasia, includinge: a decrease in HDL-cholesterol and total cholesterol, and an increase in triacylglycerol. These current research findings provide grounds to speculate that xanthan gum potentiated the effect of Walker 256 tumors on these plasma biochemical parameters.

The content of cytokines in the tissues was analyzed and the most significant result obtained was the increased IL-6, TNF-α, and IL-10 in the retroperitoneal adipose tissue of the XG group compared to that of the control group (C); and the increased TNF-α in the mesenteric adipose tissue of the XG group compared to that of the to C and TGX groups.

TNFα is considered as one of the most potent proinflammatory cytokines and is upregulated in intestinal inflammation [24]. Moreover, it was previously reported that TNF-α, IL-6, and MCP-1 mRNA expression increased in mesenteric fat depots, 2 days after the induction of Trinitrobenzene Sulfonic Acid (TNBS)-Induced Colitis in mice, which could be dependent on the NF-κB pathway [25].

Taken together our results and those of previous reports, corroborate findings of the clinical case study presented by Woods et al. in 2012 [3], which showed that the continuous use of xanthan gum in neonates causes the development of necrotizing enterocolitis, which is a highly inflammatory process. The levels of inflammatory endotoxins and cytokines are high in infants with necrotizing enterocolitis [26]. Our results confirm that the addition of xanthan gum to the diet promotes a proinflammatory state by elevating the proinflammatory cytokine TNFα in mesenteric and retroperitoneal adipose tissues and also by elevating the proinflammatory cytokine IL-6 in retroperitoneal adipose tissue.

Further study is necessary to analyze the intestinal inflammatory conditions after xanthan gum ingestion in rodent models to improve our knowledge of the mechanistic pathways involved in these inflammatory responses.

Consistent with this observation regarding intestinal inflammatory conditions, previous studies have shown that patients with Crohn's disease accumulate intra-abdominal fat. It suggests that mesenteric deposits could participate in the inflammatory response with increased expression of PPAR-γ and proinflammatory cytokine TNF-α [27].

It is known that the cause of inflammation in the colon is followed by increased expression of proinflammatory cytokines and substance P (SP), an important mediator of neurogenic inflammation. In the gut, SP modulates mucosal motility and permeability, in addition it has affinity with neurokinin 1 receptor (NK-1R- neurokinin) [28].

Previous studies indicate that the activation of the SP-NK1R system in mesenteric fat deposits plays a substantial role in the pathophysiology of intestinal inflammation by increasing expression of NK-1R in mouse mesentery after TNBS- induced colitis. This is consistent with other studies in inflammatory intestinal tissues in animals and humans [28].

Colitis has a significant expression of cytokines in mesenteric adipose tissue and secreted cytokines are likely to enter the circulation and systemic responses may be obtained in other tissues [28].

An increase of the anti-inflammatory cytokine IL-10 in the retroperitoneal tissue was noted, possibly as a reactive form. It is also interesting to note that inoculation with Walker tumor cells in the xanthan gum diet (TGX) group elevated IL-6 in the gastrocnemius muscle compared to that in the TC group. The IL-1β content of the gastrocnemius muscle was lower in the XG and TGX groups than in the C and TC groups. It is known that the first cytokines induced during inflammation are IL-1β, IL-2, IL-6, TNF-α and interferon gamma. The interleukins IL-1, IL-6 and TNF-α are known to produce direct metabolic effects, such as antioxidant defense, micronutrient redistribution, lipolytic action, and protein consumption by lean muscle mass [29]. This strengthens the argument that the prolonged use of xanthan gum in food alters the expression of pro-inflammatory cytokines.

## Conclusion

Overall, our results indicate that the continuous use of xanthan gum triggers a pro-inflammatory response, which promotes increased cytokine production, especially in adipose tissue. However, the addition of xanthan gum to the diet did not affect tumor development in animals inoculated with Walker 256 cells.

## Supporting information

S1 Table (XLSX)
